# Commentary: Allergen-specific IgE/total IgE ratio for food allergy diagnosis in children

**DOI:** 10.3389/fped.2026.1795404

**Published:** 2026-04-08

**Authors:** Kazim Okan Dolu

**Affiliations:** Department of Pediatric Allergy and Immunology, Kanuni Sultan Süleyman Training and Research Hospital, Istanbul, Türkiye

**Keywords:** child, clustered data, food hypersensitivity, immunoglobulin e ratio, oral food challenge

A Commentary on Allergen-specific IgE/total IgE ratio for food allergy diagnosis in children By Xu X, Liu L, Zhang Y, Zhou P, Wang P, Zhou J and Zhou W (2025). Front. Pediatr. 13:1628506. doi: 10.3389/fped.2025.1628506

## Introduction

I read the article by Xu et al. (1) with great interest. Identifying reliable *in vitro* markers to reduce the burden of oral food challenges (OFC) is a priority in pediatric allergy. However, I have identified several fundamental statistical concerns that, in my view, may affect the validity of the primary conclusions.

### Analysis

#### The independence problem (pseudoreplication)

The most striking issue is the mismatch between the number of subjects (*n* = 83) and the number of challenges (*n* = 209). It appears that each challenge was treated as an independent data point. In clinical research, multiple observations from the same child are inherently correlated. By failing to account for this clustering, the standard errors are likely underestimated, and the *p*-values may be artificially small. Without employing Generalized Estimating Equations (GEE) or mixed-effects models to adjust for within-subject correlation, the reported diagnostic associations remain statistically questionable.

#### Claims of superiority vs. statistical reality

The authors conclude that the sIgE/tIgE ratio is “better” than sIgE alone for the total sample and egg white groups. However, their own ROC analysis—as documented in the results and Figure 1—states that there was no significant difference between the AUCs of the two methods. If the difference between two diagnostic markers is not statistically significant, one cannot definitively claim the superiority of one over the other. This creates a contradiction between the data presented and the final inference.

#### Model overfitting and multicollinearity

In Table 3, the authors include both sIgE and the sIgE/tIgE ratio within the same multifactorial model. Since the ratio is a direct mathematical derivative of sIgE, these variables are highly collinear. Such multicollinearity can make the regression model unstable and may explain why sIgE loses its significance only when the ratio is introduced into the model.

## Conclusion

I believe these methodological issues, particularly the lack of adjustment for clustered data and the interpretation of non-significant AUC differences, warrant further clarification. A corrected analysis is essential to ensure that the proposed cut-off of 10.42 can be safely and accurately implemented in clinical practice.

## References

[1] Xu X Liu L Zhang Y Zhou P Wang P Zhou J Allergen-specific IgE/total IgE ratio for food allergy diagnosis in children. Front. Pediatr. (2025) 13:1628506. 10.3389/fped.2025.1628506 41181177 PMC12575310

